# Sensory Polymeric Foams as a Tool for Improving Sensing Performance of Sensory Polymers

**DOI:** 10.3390/s18124378

**Published:** 2018-12-11

**Authors:** Blanca S. Pascual, Saúl Vallejos, Cipriano Ramos, María Teresa Sanz, José A. Reglero Ruiz, Félix C. García, José M. García

**Affiliations:** 1 Departamento de Química, Facultad de Ciencias, Universidad de Burgos, Plaza de Misael Bañuelos s/n, 09001 Burgos, Spain; bspascual@ubu.es (B.S.P.); svallejos@ubu.es (S.V.); fegarcia@ubu.es (F.C.G.); 2 Departamento de Biotecnología y Ciencia de los Alimentos, Área de Ingeniería Química, Facultad de Ciencias, Universidad de Burgos, Plaza de Misael Bañuelos s/n, 09001 Burgos, Spain; Ciprianorr@ubu.es (C.R.); tersanz@ubu.es (M.T.S.)

**Keywords:** sensory films, microcellular polymer, ScCO_2_ foaming, mercury detection

## Abstract

Microcellular sensory polymers prepared from solid sensory polymeric films were tested in an aqueous Hg(II) detection process to analyze their sensory behavior. First, solid acrylic-based polymeric films of 100 µm thickness were obtained via radical copolymerization process. Secondly, dithizone sensoring motifs were anchored in a simple five-step route, obtaining handleable colorimetric sensory films. To create the microporous structure, films were foamed in a ScCO_2_ batch process, carried out at 350 bar and 60 °C, resulting in homogeneous morphologies with cell sizes around 5 µm. The comparative behavior of the solid and foamed sensory films was tested in the detection of mercury in pure water media at 2.2 pH, resulting in a reduction of the response time (RT) around 25% and limits of detection and quantification (LOD and LOQ) four times lower when using foamed films, due to the increase of the specific surface associated to the microcellular structure.

## 1. Introduction and Objectives

Limit of detection (LOD), limit of quantification (LOQ) and response time (RT) are three key parameters for describing the behavior of chemosensors, and they are of special relevance for solid sensory polymers, e.g., sensory films, since their specific surface of these solids is low and, at the same time, the diffusion of target chemicals into the dense polymer structure is governed by Fick’s law and by their solubility. Sensory polymeric films, specifically colorimetric chemosensory materials, are highly interesting analytical tools because they are inexpensive, they can be managed easily and can be used in situ by unskilled personnel to quantify target species in gas phase or in solution [1,2].

For this reason, it is essential to find ways for improving the performance of polymer chemosensors, in terms of the previously mentioned parameters (LOQ, LOD and RT), maintaining their chemical and physical properties without losing the advantage of detecting target species through simple processes, i.e., by putting into contact a small piece of polymer film with the measuring medium. One possible approach to reduce the LOQ, LOD and RT parameters would be an increase of the specific surface throughout the formation of a microcellular morphology, then enhancing the diffusion rate of the aqueous solution into the material, keeping the manageability of the solid sensory films.

Cellular materials, specifically polymeric foams, have been widely analyzed during the last decades, due to their combination of being low-weight with mechanical and thermal properties. We can cite the classical work of Gibson and Ashby [3] and more recent approaches published by Marsavina et al. [4,5] and Linul et al. [6,7], in which the relation between cellular structure and different properties of cellular materials are deeply investigated. In the last years, the sensory properties of cellular materials have gained a lot of attention, and different research works have been published employing porous materials in sensing applications. For example, Hwang et al. developed micro-resonators based on porous nanowires [8], Kumeria et al. presented colorimetric sensors using mesoporous silicon crystals [9], Jiang et al. prepared humidity sensors employing porous polymeric microspheres [10], and Lee et al. prepared fluorescent molecular-scale porous polymeric sensory films for the detection of volatile organic compounds [11]. It is also important to remark that the enhanced sensitivity of foams for analyte detection has also been reported by Wang et al. [12], employing the sensing characteristics of polyurethane foams in amine detection, or the classical work of Park et al., in which porous polymeric films are tested in humidity-sensing applications [13]. Luo et al. [14] described the fabrication of a pressure-sensitive array based on polydimethylsiloxane porous substrate, and a very interesting recent work presented by Liu et al. [15] presented the use of copper foams in glucose-sensing applications.

In parallel, our group has great experience in the fabrication and characterization of colorimetric polymer-based sensory films, which show the ability to detect different target species [16,17]. Recently, we have also investigated the foamability of these sensory films using the direct and clean ScCO_2_ foaming process [18,19,20], obtaining very promising results [21]. Compared to other experimental procedures that obtain microporous structures based on chemical reactions, ScCO_2_ foaming is considered a “green” process, using an inert and non-expensive gas which does not interact chemically with the polymers, simplifying greatly the process and offering the possibility of controlling the porous morphology in terms of the supercritical conditions.

Bearing all these ideas in mind, we decided to verify the previous hypothesis taking advantage of the foamability of our sensory films, selecting a specific application which has also been deeply analyzed previously in our group, to compare the sensing characteristics of solid and ScCO_2_ foamed microcellular films. It is well known that sensing of heavy metal cations has been investigated in the last decade, and specifically mercury detection has become a fundamental research line due to its toxicity. Different works have been published concerning this topic, using different sensory systems. For example, Yang et al. [22] presented a colorimetric nanosensor based on gold nanoparticles for the detection of Hg(II) ions; Zhang and Leng [23] reported the development of fluorescent sensors for Hg(II) cations based on a coumarin–rhodamine system; and Xiao et al. [24] described a portable smartphone device for the detection of mercury contamination. Following this research line, we selected a trusted colorimetric sensory polymeric film for detecting Hg(II) in water solution [25], which has already been tested previously in our group, increasing its specific surface in a single-batch ScCO_2_ foaming process.

After ScCO_2_ foaming, sensing of Hg(II) was carried out employing both solid and foamed samples as colorimetric sensory films, adding dithizone motifs as moieties (**DZ**), which were chemically anchored to the sensory film. The film was green before the sensing process, and it turned red upon entering into contact with Hg(II), using the color variation to quantify the concentration of Hg(II) through the RGB parameters of digital pictures of the sensory film taken with a smartphone. The sensing characteristics of non-foamed samples (dense films) and foamed samples were compared by detecting Hg(II) in aqueous solution, analyzing specifically the response time (RT), the limit of detection (LOD) and the limit of quantification (LOQ).

## 2. Materials and Methods

**Scheme 1:**
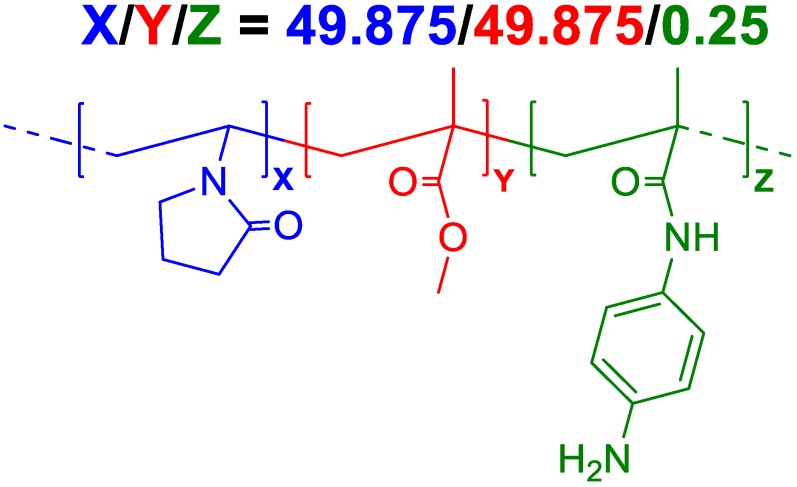
Chemical structure of the pre-sensory film.

The dense pre-sensory film of 115 µm thickness was prepared via the radical copolymerization process described in detail in our previous work [25]. 1-Vinyl-2-pyrrolidone (**VP**) was copolymerized with methyl methacrylate (**MMA**) and 4-vinylaniline (**VA**) with molar percentages of 49.875(**VP**)/49.875(**MMA**)/0.250(**VA**), via radical copolymerization process, using 2,2′-azobis(2-methylpropionitrile) (**AIBN**) at 1 wt% molar as a thermal radical initiator. Afterwards, the bulk radical polymerization reaction was carried out in a silanized glass mould in an oxygen-free atmosphere at 60 °C overnight. The chemical structure of the pre-sensory film is presented in Scheme 1.

**Scheme 2:**
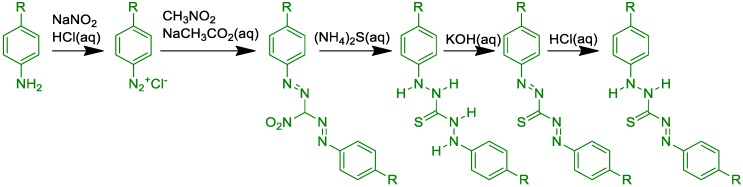
Five-step route to prepare the sensory film with dithizone-derivative moieties (Reproduced from ref. [25] with permission from The Royal Society of Chemistry).

In the second step, the pre-sensory film was transformed into the sensory film by anchoring the **DZ** motifs to the amino groups of the polymer structure in a five consecutive solid-state reactions procedure, which is also described in our previous work [25], and is depicted in Scheme 2. 

**Scheme 3:**
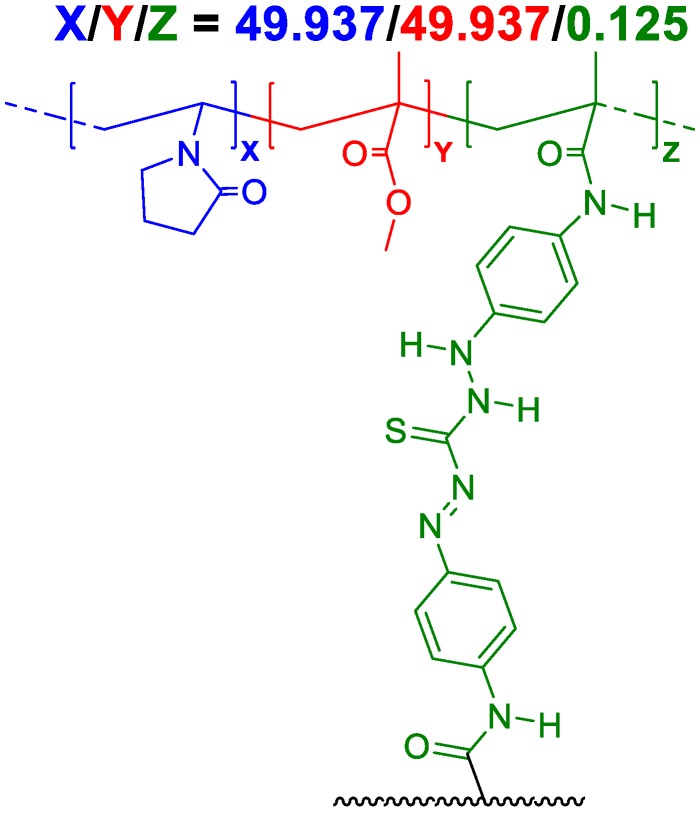
Chemical structure of the sensory film.

The sensory film was prepared by immersion of the pre-sensory film in five different and consecutive aqueous reaction media, giving rise to consecutive solid-state reactions. During this process, **DZ**-derivative motifs were obtained, which acted as cross-linking residues. In the first step, the pre-sensory film was immersed for 30 min in a solution of 250 mL of water, 25 mL of HCl and 1 g of NaNO_2_. The amino functional groups of the anchorage monomer *N*-(4-aminophenyl) methacrylamide) react to produce a diazonium salt. Rapidly, and without washing the material, the film was immersed for 90 min in a solution of 250 mL of water, 10 g of sodium acetate and 1 mL of nitromethane, which induced the cross-linking of the polymer by the formation of bridges of the 2,2′-(nitromethylene) bis(1-phenyldiazene) derivative. In the third step, 2-(2-(2-phenylhydrazinecarbonothioyl) hydrazinyl) benzene motifs were obtained upon immersion of the film in a solution of 250 mL of water and 30 mL of aqueous solution of (NH_4_)_2_S (20%) for 90 min. Then, the film was washed thoroughly with water. The fourth step consisted of a deprotonation process using 250 mL aqueous solution of KOH (4%) for 30 min at 50 °C to obtain the bis (phenyldiazenyl) methanethione intermediate. In the last step, the **DZ**-derivative moieties were obtained by immersing the membrane in 250 mL of aqueous solution of HCl (4%) for 1 min, obtaining the sensory film. The final chemical structure of the sensory film is presented in Scheme 3. 

**Figure 1:**
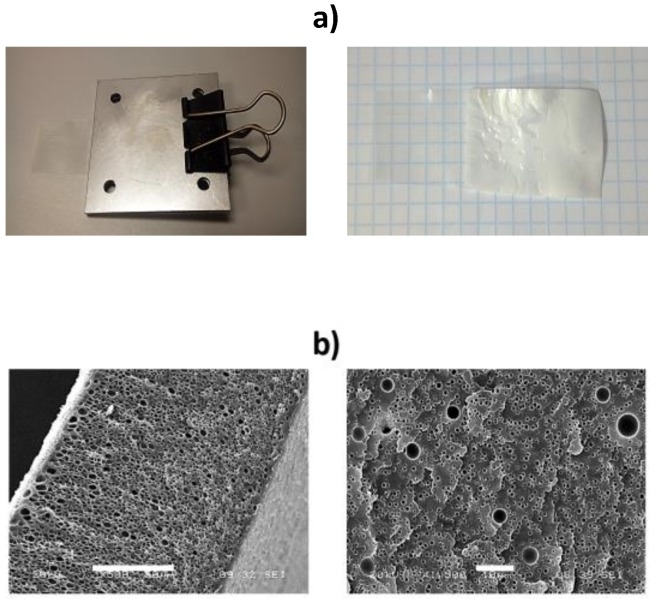
(**a**) Experimental setup to obtain a film with solid and foamed regions: Steel mold (**left**) and film after ScCO_2_ processing with the two regions (**right**); (**b**) SEM micrographs of the foamed region of the sensory film: Cross-section (**left**, bar scale 50 µm) and surface (**right**, bar scale 10 µm).

Finally, sensory films were foamed using ScCO_2_ in a single-step batch process. Films were cut in pieces of 70 × 35 mm^2^, and were partially sandwiched between two steel plates of 50 × 50 mm^2^ and 2 mm thickness. This experimental setup is specifically designed to confine the gas between the steel plates during the depressurization process, limiting the gas diffusion outside the membrane, which is reduced significantly due to the presence of the steel plate, then promoting the cell formation. In order to compare the solid and foamed materials, half of the surface of the film was left outside the mold during the ScCO_2_ process, thus obtaining samples with both solid and foamed regions from an unique starting membrane. This experimental setup, to produce microcellular polymeric films using ScCO_2_, has been recently described by our group [21] and previously by Siripurapu et al. [18,19]. During ScCO_2_ foaming, samples were saturated with ScCO_2_ in a high-pressure reactor at 35 MPa and 60 °C for 24 h, and then depressurized quickly in about 10 s. Figure 1 shows photographs of the experimental set-up and the obtained films together with a couple of SEM micrographs of the foamed region (surface and cross-section). A homogeneous cell structure in the cross-section with an average cell size around 5 µm was observed. On the other hand, on the surface of the foamed region of the film, the porous structure presented an irregular cell size distribution, showing smaller cell sizes (around 1 µm radii) with isolated larger pores (about 5 µm radii).

## 3. Results and Discussion

The sensing process was carried out using an aqueous solution of Hg(II) (913 ppm, Hg(NO_3_)_2_, pH 2.2 buffered: KCl/HCl). These conditions were selected for two main reasons: First, the Hg(II) detection must be carried out in acid media, due to the interference problems observed when using a basic media for the Hg(II) sensing. Secondly, we employed a high Hg(II) concentration in order to reduce the detection time and capture in video the whole process. It is possible to use lower concentrations (in the range of 5 ppm) which are close to the concentration of a real application, but in this case, the detection time would increase up to several hours. Two square samples of 20 × 20 mm^2^ were immersed in the solution, one corresponding to the foamed region and the other one extracted from the solid region of the film. Additionally, a plastic piece was placed between both samples and was used as a blank reference in the RGB (R = red, G = green, B = blue) calculations. The whole sensing process was captured in video for 60 min, then extracting different photographs of the color evolution of the discs at different times to perform the RGB analysis. A total number of 26 photographs were extracted from the video, following the next distribution: During the first 20 min, one photograph each minute was taken. Then, from 20 min to 30 min, one photograph each 5 min, and finally, from 30 min to 60 min, one photograph each 10 min.

**Figure 2:**
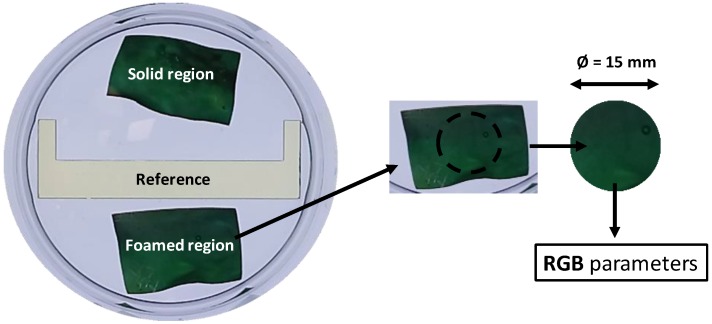
Detection process: Picture of the Hg(II) solution with the immersed films (**left**), and circular section selected from the picture of the film to obtain the RGB parameters (**right**).

RGB analysis was carried out with a conventional processing image software. To avoid border effects, a circular area of 15 mm diameter was selected from the picture of each film. In the case of the blank material, a square section of 10 mm^2^ was delimited. Calculations of RGB parameters of the discs were normalized using the RGB parameters of the reference material, which did not vary during all the sensing processes. Figure 2 shows the experimental setup employed in the Hg(II) detection process. Each of the images (solid region, foamed region and reference material) was analyzed using a conventional image processing software to determine the RGB parameters at different detection times. The variation along time of color is clearly visible to the naked eye for both regions (solid and foamed), and can be graphically seen following the parameters R, G and B of the images, as is shown in Table 1, in which we present the most representative pictures of both types of discs taken during the detection process, together with the numerical RGB parameters obtained from each image.

**Figure 3:**
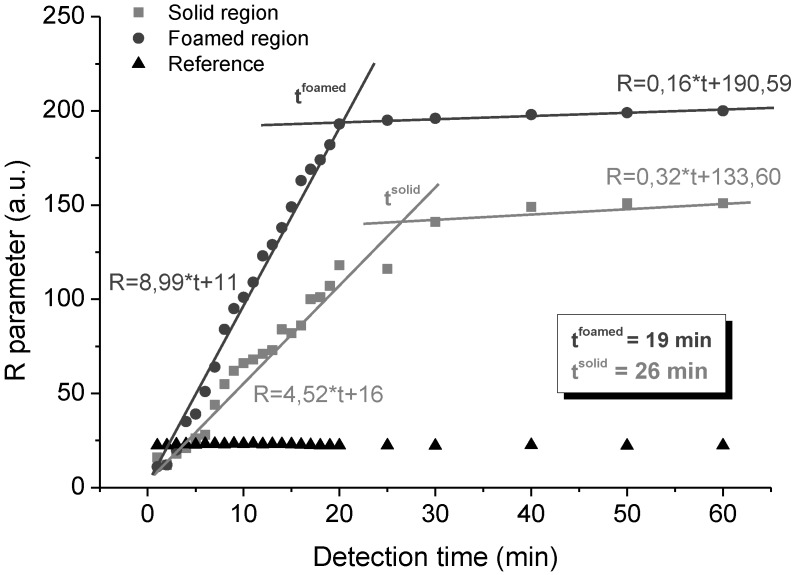
Evolution of the red parameter (R) of images of sensory films, and reference along time immersed in the aqueous solution of Hg(II) (913 ppm, Hg(NO_3_)_2_, pH 2.2 buffered: KCl/HCl).

As can be seen in Table 1, the color variation is more intense and also faster when using foamed films, especially in detection times above 15 min. Quantification of this visual evidence can be carried out through the variation of the red (R) parameter, which is presented in Figure 3, determining the detection time of the solid and foamed regions of the sensory film. An estimation of the detection time can be carried out using two different linear fittings of the red value evolution. Two different zones are considered in each curve to perform the linear fitting, which is delimited by the saturation value of the red parameter (detection time of foamed (t_foamed_) and solid (t_solid_) sensory films). In our case, detection time was reduced around 25%, lowering the value from 26 min in solid films (t_solid_) to 19 min in foamed films (t_foamed_). On the other hand, no great differences were observed in the G (green) and B (blue) parameters, as is shown in Figures S1 and S2 of Section S1.1 of the Electronic Supplementary Information (ESI) file.

Moreover, the red parameter values of the foamed discs are higher through all the detection processes than the values measured for the solid disc. The saturation value of the red parameter for the solid disc is around 150 arbitrary units (a.u.), whereas for the foamed disc this value reaches 200 a.u. Accordingly, the color change is much more visible and is more easily detectable by the naked eye when foamed sensory films are used (see pictures of the sensory discs in Table 1). Thus, it is concluded that both the detection time and also color intensity are improved when microcellular films are used in the detection of Hg(II) cations in aqueous solution.

Another key parameter that must be taken into account is the sensitivity of these chemosensors. For this purpose, titration curves of Hg(II) were carried out using the procedure followed in our previous work, [25] analyzing the RGB parameters of digital pictures taken to the sensory films, using a homemade retro-illumination system to standardize the illumination conditions. To clearly differentiate the sensory materials, samples were cut in squares of 5 mm a side in the case of solid samples, and in triangles of 5 mm a side for foamed samples. Samples were immersed into separate water solutions containing Hg(II) (913 ppm, Hg(NO_3_)_2_, pH 2.2, buffered: KCl/HCl). The principal component PC1 of the R and B parameters allowed for the construction of a titration curve over a range of concentrations (from 1 × 10^−7^ to 3 × 10^−6^ M), from which the LOD and LOQ values were calculated (see Table 2), using the following equations: LOD = 3.3 × SD/s and LOQ = 10 × SD/s, where SD is the standard deviation of a blank sample and s is the slope of the calibration curve in a region of low Hg(II) content. We also include in Table 2, for comparison purposes, the values of LOD and LOQ obtained in our previous work, [25] in which a solid film of the same composition was tested in similar conditions. It is observed that the foamed sensory films present values of LOD and LOQ which are four times lower than the values observed in solid sensory samples, thus confirming the relevant effect of the microcellular structure in the improvement of the performance of the material as a chemosensor. We present in Section S1.2 of the ESI the principal component analysis PC1 and the titration curve of Hg(II) from the RGB parameters calculated over the range of concentrations exposed above, for both solid and foamed films, in which the improvement of the sensing behavior when using microcellular foamed films is evident (please see Tables S1 and S2 and also Figures S3 and S4 of the ESI).

## 4. Conclusions

In short, we have enhanced the performance of sensory polymer films (in terms of detection time, limit of detection and limit of quantification) by preparing foamed microcellular structures. Solid sensory films were foamed via ScCO_2_ foaming process, and detection of Hg(II) in aqueous solution was carried out, analyzing the behavior of both solid and foamed films in terms of response time and limit of detection. Results showed that using foamed films led to a reduction in the response time of around 25%, and values of limit of detection and quantification were also improved around twofold with respect to solid films, mainly due to the increased specific surface exposed to the Hg(II) solution. This ScCO_2_ foaming process is a straightforward and green alternative procedure for physically improving the performance of known polymer chemosensors without modifying the chemistry, compared to non-environmentally clean, expensive and complicated traditional processes. Moreover, the ScCO_2_ foaming process could be easily applied to other polymer-based sensory films with good foamability.

## Figures and Tables

**Table 1:** RGB data from photographs taken from the solid and foamed regions of the sensory films after immersion for 60 min at RT in aqueous solutions (pH = 2.2, KCl–HCl) with a fixed concentration (913 ppm) of Hg(II).

Solid Region	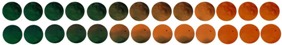									
Foamed Region										
**Time (min)**	1	2	3	4	5	10	15	20	25	30
**R (Solid)**	6	12	18	21	26	66	82	118	116	141
**R (Foamed)**	11	12	20	35	39	101	149	193	195	196
**G (Solid)**	67	55	58	52	54	62	51	63	61	41
**G (Foamed)**	63	61	59	60	57	67	79	93	93	94
**B (Solid)**	39	34	37	38	28	36	23	30	22	19
**B (Foamed)**	39	42	42	39	33	35	32	28	28	31

**Table 2:** Limit of detection (LOD) and limit of quantification (LOQ) values determined from titration curves of Hg(II) for solid and foamed films, compared to our previous results taken from reference [25].

Sensory Film	LOD, ppb	LOQ, ppb
Solid (this work)	1.1	3.4
Foamed (this work)	0.3	0.9
Solid, Vallejos et al. [25]	1.6	4.8

## Supplementary Materials

The following are available online at http://www.mdpi.com/1424-8220/18/12/4378/s1; Figure S1: Evolution of the green parameter (G) of images of sensory films along time upon entering into contact with a water solution of Hg(II) (913 ppm), Figure S2: Evolution of the blue parameter (B) of images of sensory films along time upon entering into contact with a water solution of Hg(II) (913 ppm), Figure S3: Variation of the PC1 vs. the logarithm of the Hg(II) concentration for foamed discs. Upon fitting with a two-degree polynomial, the mercury concentration in the test sample was calculated, Figure S4: Variation of the PC1 vs. the logarithm of the Hg(II) concentration for solid discs. Upon fitting with a two-degree polynomial, the mercury concentration in the test sample was calculated, Table S1: Hg(II) concentrations, RGB parameters and PC1 of each foamed disk. Values in red were neglected for plotting the titration curves, Table S2: Hg(II) concentrations, RGB parameters and PC1 of each solid disk. Values in red were neglected for plotting the titration curves.
