# Supplementary material for: Sensory Polymeric Foams as a Tool for Improving Sensing Performance of Sensory Polymers
Source: Sensors (Basel). 2018 Dec 11;18(12):4378. doi: 10.3390/s18124378 (PMC6308416; doi:10.3390/s18124378)
Supplement: Supplementary file 1 [file sensors-18-04378-s001.pdf]

Electronic Supporting information for:

Sensory polymeric foams as a tool for improving sensing performance of sensory polymers

Blanca S. Pascual,[a] Saúl Vallejos,[a] Cipriano Ramos,[b] María Teresa Sanz,[b] José A. Reglero Ruiz,*,[a] Félix C. García[a] and José M. García[a]

[a] Departamento de Química, Facultad de Ciencias, Universidad de Burgos, Plaza de Misael Bañuelos s/n, 09001 Burgos, Spain. Tel: +34 947 258 085; Fax: +34 947 258 831.

[b] Departamento de Biotecnología y Ciencia de los Alimentos, Área de Ingeniería Química, Facultad de Ciencias, Universidad de Burgos, Plaza de Misael Bañuelos s/n, 09001 Burgos, Spain. Tel: +34 947 258 085; Fax: +34 947 258 831.

* Corresponding author email: jareglero@ubu.es

Table of contents

S1. Colorimetric detection of Hg(II) 2

S1.1 Green (G) and Blue (B) parameters variation of images of sensory discs and the reference material.. 2

S1.2 Titration of Hg(II) examining the analysis of the RGB parameters of both foamed and solid discs using a digital picture.. 3

S1.2.1 Foamed discs 3

S1.2.2 Solid discs 4

S1. Colorimetric detection of Hg(II)

S1.1 Green (G) and Blue (B) parameters variation of images of sensory discs and the reference material

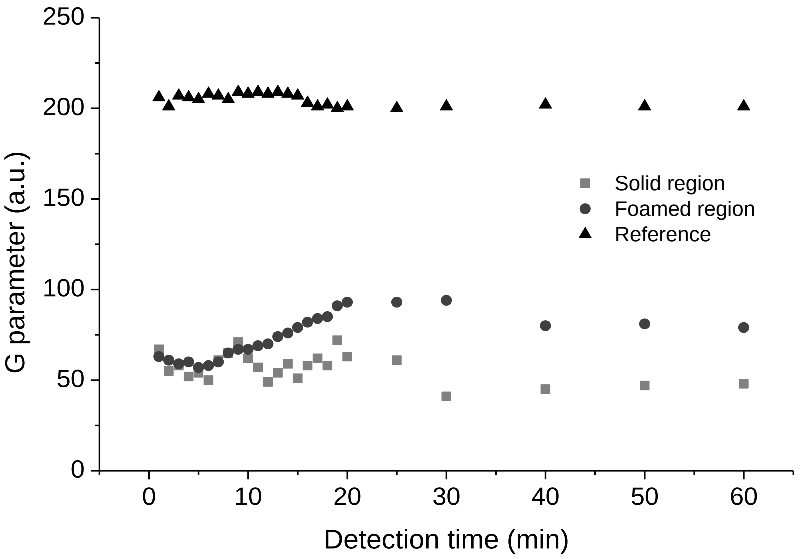

Figure S1. Evolution of the green parameter (G) of images of sensory films along time upon entering into contact with a water solution of Hg(II) (913 ppm).

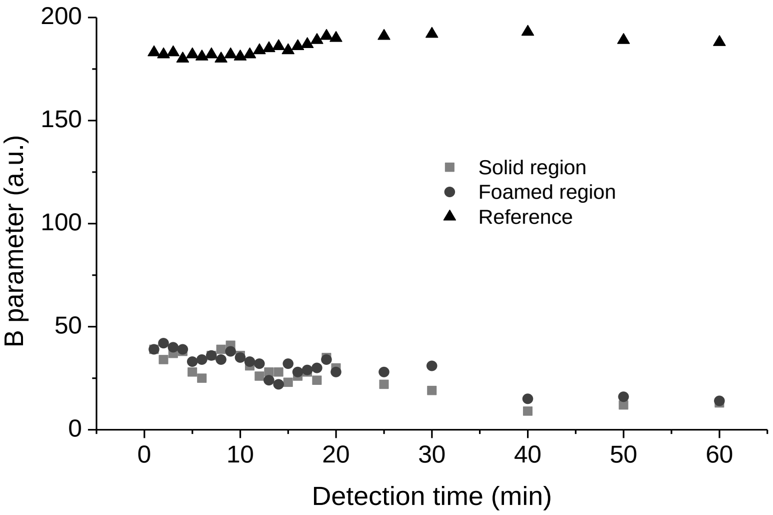

Figure S2. Evolution of the blue parameter (B) of images of sensory films along time upon entering into contact with a water solution of Hg(II) (913 ppm).

S1.2 Titration of Hg(II) examining the analysis of the RGB parameters of both foamed and solid discs using a digital picture

S1.2.1 Foamed discs

Table S1. Hg(II) concentrations, RGB parameters and **PC1** of each foamed disk. Values in red were neglected for plotting the titration curves.

Hg(II), ppb	Log [Hg(II), ppb]	R	G	B	PC1 (R&G)
**0**	**n/a**	**215**	**215**	**191**	**Not used**
1,00E-07	-7	16	28	32	-0,5770
5,00E-07	-6,3010	14	31	32	-0,5666
1,00E-06	-6	18	28	35	-0,5596
**2,00E-06**	**-5,6989**	**13**	**22**	**27**	**Not used**
3,00E-06	-5,5228	18	32	33	-0,5226

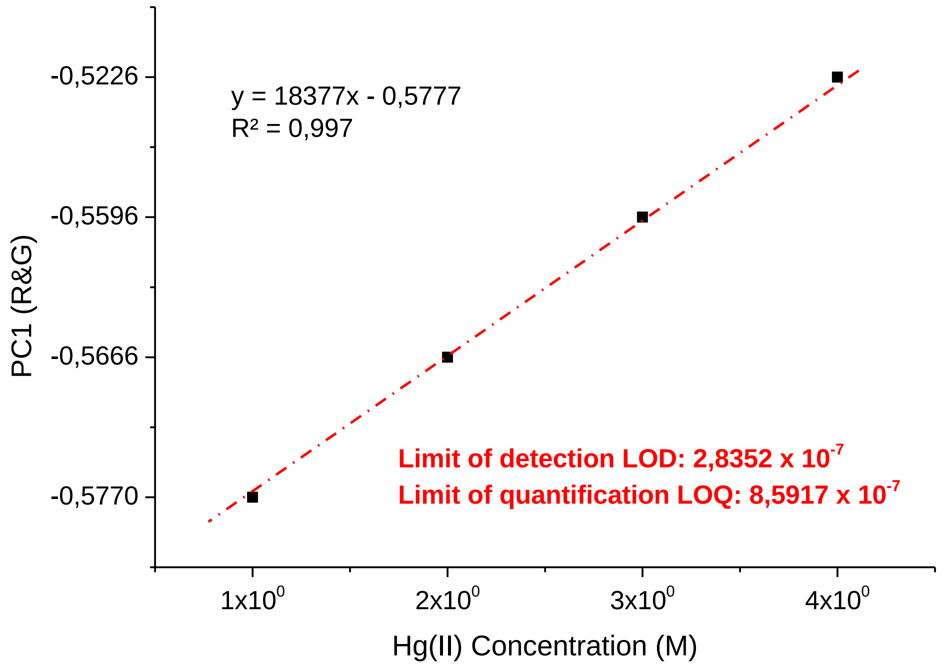

Figure S3. Variation of the **PC1** *vs* the logarithm of the Hg(II) concentration. Upon fitting with a two-degree polynomial, the mercury concentration in the test sample was calculated.

S1.2.2 Solid discs

Table S2. Hg(II) concentrations, RGB parameters and **PC1** of each solid disk. Values in red were neglected for plotting the titration curves.

Hg(II), ppb	Log [Hg(II), ppb]	R	G	B	PC1 (R&G)
**0**	**n/a**	**39**	**113**	**98**	**Not used**
1,00E-07	-7	20	100	73	-0,7688
5,00E-07	-6,3010	28	95	80	-0,2183
1,00E-06	-6	28	97	80	-0,3501
**2,00E-06**	**-5,6989**	**22**	**83**	**66**	**Not used**
3,00E-06	-5,5228	88	86	91	2,0352

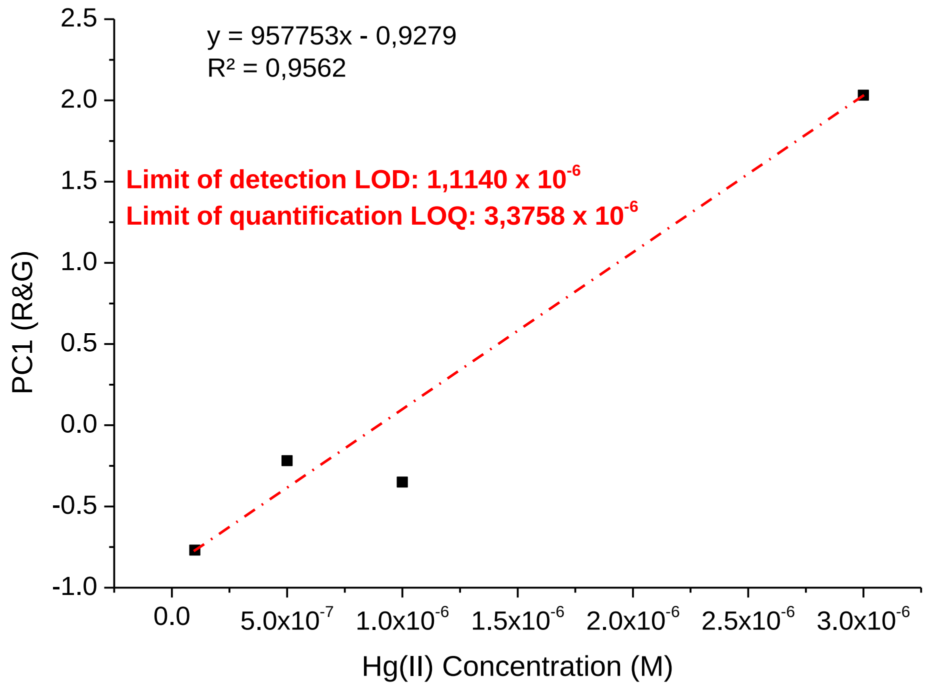

Figure S4. Variation of the **PC1** *vs* the logarithm of the Hg(II) concentration. Upon fitting with a two-degree polynomial, the mercury concentration in the test sample was calculated.
